# Mosaic Loss of Chromosome Y Is Associated With Functional Outcome After Ischemic Stroke

**DOI:** 10.1161/STROKEAHA.123.043551

**Published:** 2023-07-19

**Authors:** Malin Dorvall, Annie Pedersen, Jan P. Dumanski, Martin Söderholm, Arne G. Lindgren, Tara M. Stanne, Christina Jern

**Affiliations:** 1 Department of Laboratory Medicine, Institute of Biomedicine, Sahlgrenska Academy, University of Gothenburg, Sweden (M.D., A.P., T.M.S., C.J.).; 2 Department of Clinical Genetics and Genomics, Region Västra Götaland, Sahlgrenska University Hospital, Gothenburg, Sweden (A.P., C.J.).; 3 Department of Immunology, Genetics and Pathology and Science for Life Laboratory, Uppsala University, Sweden (J.P.D.).; 4 3P-Medicine Laboratory, Medical University of Gdańsk, Poland (J.P.D.).; 5 Department of Clinical Sciences Lund, Neurology, Lund University, Sweden (M.S., A.G.L.).; 6 Department of Neurology, Skåne University Hospital, Lund and Malmö, Sweden (M.S., A.G.L.).

**Keywords:** brain infarction, brain ischemia, chromosomes, genetics, humans, male, stroke

## Abstract

**BACKGROUND:**

Mosaic loss of chromosome Y (LOY) is associated with cardiovascular and neurodegenerative diseases in men, and genetic predisposition to LOY is associated with poor poststroke outcome. We, therefore, tested the hypothesis that LOY itself is associated with functional outcome after ischemic stroke.

**METHODS:**

The study comprised male patients with ischemic stroke from the cohort studies SAHLSIS2 (Sahlgrenska Academy Study on Ischemic Stroke Phase 2; n=588) and LSR (Lund Stroke Register; n=735). We used binary logistic regression to analyze associations between LOY, determined by DNA microarray intensity data, and poor 3-month functional outcome (modified Rankin Scale score, >2) in each cohort separately and combined. Patients who received recanalization therapy were excluded from sensitivity analyses.

**RESULTS:**

LOY was associated with about 2.5-fold increased risk of poor outcome in univariable analyses (*P*<0.001). This association withstood separate adjustment for stroke severity and diabetes in both cohorts but not age. In sensitivity analyses restricted to the nonrecanalization group (n=987 in the combined cohort), the association was significant also after separate adjustment for age (odds ratio, 1.6 [95% CI, 1.1–2.4]) and when additionally adjusting for stroke severity and diabetes (odds ratio, 1.6 [95% CI, 1.1–2.5]).

**CONCLUSIONS:**

We observed an association between LOY and poor outcome after ischemic stroke in patients not receiving recanalization therapy. Future studies on LOY and other somatic genetic alterations in larger stroke cohorts are warranted.

Somatic genetic alterations are an emerging field in research for stroke and neurological diseases. Mosaic loss of chromosome Y (LOY) is the most common somatic alteration, and the proportion of cells with LOY increases with age.^1^ LOY occurs most frequently in hematopoietic tissue but also occurs in the brain.^1,2^ LOY in peripheral blood cells is independently associated with major adverse health outcomes in men, such as cardiovascular disease, diabetes, and Alzheimer disease.^3,4^ There is an increased proportion of cells with LOY in postmortem brain tissue from patients with neurodegenerative disorders compared with controls.^1,2^ We found that a polygenic risk score for LOY^5^ is associated with poor poststroke outcome.^6^ Based on this background, we hypothesized that LOY itself is associated to poor ischemic stroke outcome.

## METHODS

The data that support this study are available upon reasonable request.

### Study Population and Outcome

This study comprised male ischemic stroke patients from an exploratory cohort (SAHLSIS2 [Sahlgrenska Academy Study on Ischemic Stroke Phase 2]; n=588) and an external validation cohort (LSR [Lund Stroke Register]; n=735). Written informed consent was obtained, and studies were approved by the appropriate ethical review boards. Stroke severity was assessed by the National Institutes of Health Stroke Scale at admission. Data on death and dependency 3 months after index stroke were retrieved from the Swedish National Quality Register, Riksstroke, and modified Rankin Scale scores were estimated as described.^7^ Good functional outcome was defined as modified Rankin Scale score of 0 to 2 and poor as modified Rankin Scale score of 3 to 6. Our study fulfills the STROBE (Strengthening the Reporting of Observational Studies in Epidemiology) checklist guidelines. Further cohort details can be found in the Supplemental Material.

### Analysis of LOY

DNA from peripheral blood collected at index stroke was genotyped using an Illumina Infinium microarray. Normalized intensity data and the MADloy software^8^ were used to calculate the median log R ratio of the male specific part of the Y chromosome, and the cut point to define LOY was set to <−0.15.^9^

### Statistical Analysis

We analyzed associations between LOY and outcome by binary logistic regression analyses. As covariates, we selected age and diabetes because they are associated with both LOY and ischemic stroke outcome^3,6,10^ and the National Institutes of Health Stroke Scale as it is a strong predictor of poststroke outcome.^10^ Separate models for each covariate were used to minimize data missingness. Analyses with all covariates were also performed after imputation for missing values. Sensitivity analyses excluding patients receiving recanalization therapy, that is, intravenous thrombolysis or endovascular therapy, were performed as especially endovascular therapy in many cases greatly improves outcome. Two-tailed *P*<0.05 was considered significant.

## RESULTS

Baseline characteristics are shown for all patients with stroke in the Table and for patients with and without recanalization therapy in Table S1. In SAHLSIS2, LOY was significantly associated with poor outcome in univariable analysis, and this association withstood adjustment for the National Institutes of Health Stroke Scale and diabetes but not age (Figure [A]). These results were replicated in LSR, and similar associations were observed in the combined cohort (Figure [A]).

**Table.**
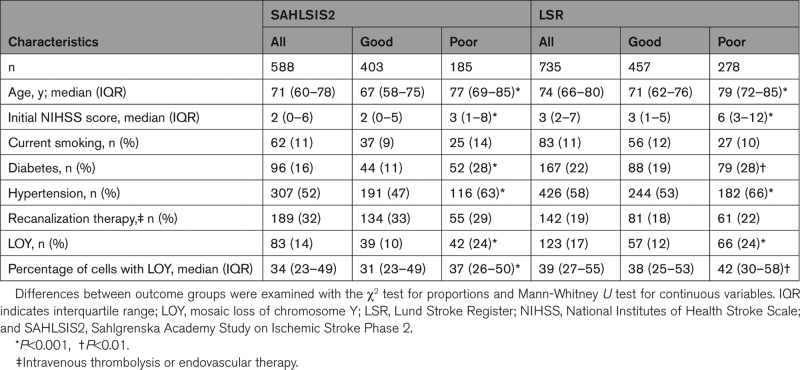
Baseline Characteristics for All Male Ischemic Stroke Patients and the Groups With Good (Modified Rankin Scale Score, ≤2) and Poor (Modified Rankin Scale Score, >2) Outcome at 3 Months in SAHLSIS2 and LSR

**Figure.**
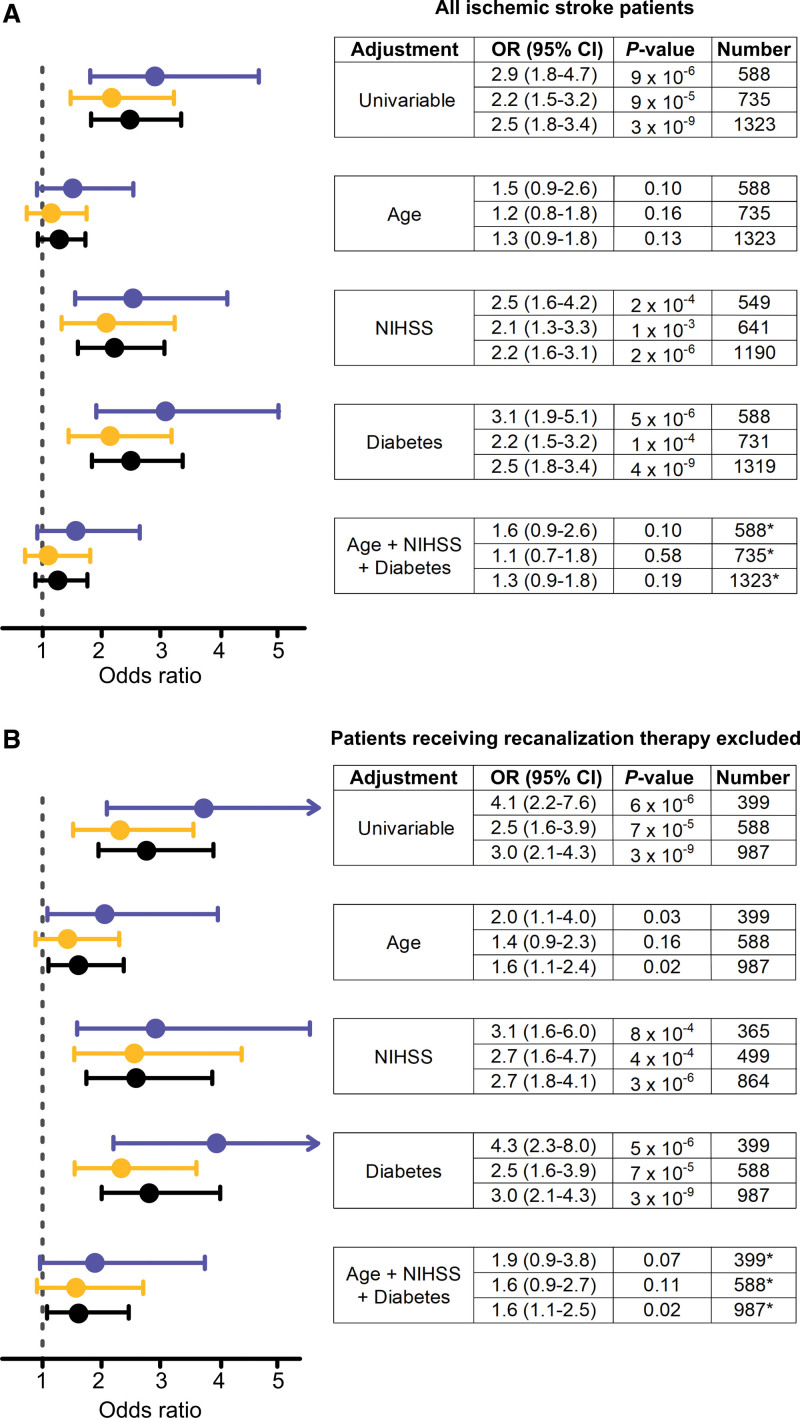
**Forest plots for the associations between mosaic loss of chromosome Y (LOY) and poor outcome after ischemic stroke.** Odds ratios (ORs) and 95% CIs for poor outcome (modified Rankin Scale score, >2) for male ischemic stroke patients with LOY compared with patients without. Results from univariable regression analyses and after adjusting for covariates as indicated for SAHLSIS2 (Sahlgrenska Academy Study on Ischemic Stroke Phase 2; blue), LSR (Lund Stroke Register; yellow), and combined cohorts (black). **A**, All patients. **B**, Excluding patients receiving recanalization therapy. NIHSS indicates National Institutes of Health Stroke Scale. *Missing data were imputed.

In sensitivity analyses excluding patients receiving recanalization therapy, the effect sizes were generally higher compared with the whole group (Figure [B]). These associations were significant also when adjusting for age in SAHLSIS2 and in the combined cohort and when adjusting for age, National Institutes of Health Stroke Scale, and diabetes in the combined cohort (Figure [B]).

## DISCUSSION

We found an association between LOY and poor functional outcome after ischemic stroke that was independent of age, stroke severity, and diabetes in patients not receiving recanalization therapy.

With regard to potential mechanisms, LOY in leukocytes has been shown to affect the expression of genes regulating immune surveillance.^11^ In brain tissue, LOY is enriched in microglia where it has similar effects on gene regulation.^2^ This suggests that LOY in microglia and leukocytes infiltrating the brain directly influences pathways involved in stroke recovery. It is also possible that other acquired structural chromosomal alterations and mutations that can co-occur with LOY, and reflect genomic instability, contribute to our findings. In support for such a hypothesis, we found associations for the polygenic risk score for LOY and stroke outcome in both sexes,^6^ and similar findings have been made for other phenotypes.^5^ In this context, it is noteworthy that LOY also coexists with mutations causing clonal hematopoiesis of indeterminate potential,^3^ which was recently reported to be more prevalent in young cryptogenic stroke compared with controls.^12^

Strengths of our study are that it comprises consecutive patients from 2 independent cohorts and that we used a microarray with good coverage over the Y chromosome. Nonetheless, this study has a limited sample size and lacks data on prestroke modified Rankin Scale and cognitive status. Further larger studies are warranted to investigate associations in different strata based on for instance ischemic stroke subtype, the presence of vascular risk factors, medical treatments, cardiovascular disease, and other comorbidities. It is also of note that in this first exploratory study, we chose to define LOY using 1 commonly used cutoff for genotype microarray data,^9^ but there are alternative ways of analyzing LOY, which should be explored in future studies.

In conclusion, we found an independent association between LOY and poor outcome in the nonrecanalization therapy group, indicating that LOY may influence the natural course of recovery after ischemic stroke in men. Future studies on LOY and other acquired genetic variants in larger stroke cohorts are warranted, as well as mechanistic studies to disentangle the possible impact of LOY on stroke recovery.

## ARTICLE INFORMATION

### Acknowledgments

The authors thank the study participants; Riksstroke, BiobankSweden, the SNP&SEQ Technology Platform, National Genomics Infrastructure, Science for Life Laboratory, Uppsala; and especially Tomas Axelsson for normalizing our genotypic data. They also thank Sanna Abrahamsson, Bioinformatics and Data Centre, Sahlgrenska Academy, for bioinformatic support, and Daniil Sarkisyan, Uppsala University, for providing input on an earlier version of this article.

### Sources of Funding

This study was supported by the Swedish Heart and Lung Foundation (Drs Jern, Lindgren, and Dumanski); Swedish Research Council (Drs Jern, Lindgren, and Dumanski); Swedish state under the agreement between the Swedish government and the county councils, the ALF agreement (Drs Jern and Lindgren); Gothenburg Foundation for Neurological Research (Dr Pedersen); King Gustaf V:s and Queen Victoria Foundation (Dr Jern); Sparbanksstiftelsen Färs och Frosta, Fremasons Lodge of Instruction Eos in Lund (Dr Lindgren); Lund University (Dr Lindgren); Region Skåne (Dr Lindgren); NIH (Dr Lindgren); and the Swedish Cancer Society (Dr Dumanski).

### Disclosures

Dr Dumanski is a cofounder and shareholder in Cray Innovation AB. Dr Lindgren reports personal fees from AstraZeneca, Bayer, Bristol Myers Squibb, Novo Nordisk, Pfizer, and Portola Pharmaceuticals. The other authors report no conflicts.

### Supplemental Material

Supplemental Methods

Checklist

Table S1

## Supplementary Material
