# Supplementary material for: Mosaic Loss of Chromosome Y Is Associated With Functional Outcome After Ischemic Stroke
Source: Stroke. 2023 Jul 19;54(9):2434–7. doi: 10.1161/STROKEAHA.123.043551 (PMC10453343; doi:10.1161/STROKEAHA.123.043551)
Supplement: Supplementary file 2 [file str-54-2434-s002.pdf]

SUPPLEMENTAL MATERIAL

Stroke

Mosaic loss of chromosome Y is associated with functional outcome after ischemic stroke

Malin Dorvall, MSc[1], Annie Pedersen, MD, PhD[1,2], Jan P. Dumanski, PhD[3,4], Martin Söderholm, MD, PhD [5,6] Arne G. Lindgren, MD, PhD[5,6], Tara M. Stanne, PhD[1*] and Christina Jern, MD, PhD[1,2*]

*Equal contribution to the work.

[1]Department of Laboratory Medicine, Institute of Biomedicine, Sahlgrenska Academy, University of Gothenburg, Gothenburg, Sweden
[2]Region Västra Götaland, Sahlgrenska University Hospital, Department of Clinical Genetics and Genomics, Gothenburg, Sweden
[3]Department of Immunology, Genetics and Pathology and Science for Life Laboratory, Uppsala University, Uppsala, Sweden
[4]3P-Medicine Laboratory, Medical University of Gdańsk, Gdańsk, Poland
[5]Department of Clinical Sciences Lund, Neurology, Lund University, Lund, Sweden
[6]Department of Neurology, Skåne University Hospital, Lund and Malmö, Sweden

Corresponding author: Malin Dorvall. E-mail: malin.dorvall@gu.se

Methods
Anonymized data will be shared upon reasonable request from a qualified academic investigator as long as data transfer agrees with EU legislation on the general data protection regulation (GDPR) and with decisions by the Ethical Review Board of Sweden and the University of Gothenburg, the latter which should be regulated in a data transfer agreement.

Study populations
The ongoing Sahlgrenska Academy Study on Ischemic Stroke phase 2 *(SAHLSIS2)* and Lund Stroke Register (*LSR*) study consecutively include adult first-ever or recurrent stroke patients at stroke units at the Sahlgrenska University Hospital in Gothenburg and first-ever stroke patients from the local catchment area and hospitalized at Skåne University Hospital in Lund, respectively, both Sweden. In the present study male participants with acute ischemic stroke recruited between 2015 and 2020 in *SAHLSIS2*, and between 2013 and 2020 in *LSR*, were included. In line with the World Health Organization stroke definition, ischemic stroke was diagnosed in patients with rapidly developing clinical signs of focal disturbance of cerebral function lasting >24 hours and without hemorrhage or signs of another cause on neuroimaging. All patients included in this study underwent computed tomography (CT) and/or magnetic resonance (MR) imaging of the brain as part of the clinical routine investigation. If clinically indicated, patients were also evaluated by CT or MR angiography and perfusion measures. Patients received recanalization therapy with intravenous thrombolysis only (IVT, n=97 in *SAHLSIS2* and n=97 in *LSR*) or endovascular therapy with or without bridging IVT (n=92 in *SAHLSIS2* and n=45 in *LSR*) according to national guidelines issued by the Swedish Board of Health and Welfare in 2015, and before that according to local hospital guidelines. Data regarding these treatments were registered at each hospital and reported to national quality registers. The patients also underwent additional work-up according to national guidelines, and patients were excluded from this study if further evaluation showed another etiology of the presenting symptoms than ischemic stroke.

Written informed consent, or consultant consent from next-of-kin, was obtained prior to enrollment. The studies were approved by the respective local Regional Ethics Review Board (Etikprövningsnämnden; EPN) or the Swedish Ethical Review Authority (Etik-prövningsmyndigheten; EPM). The registration numbers for *SAHLSIS2* are EPN Dnr 823-13 and 823-13 T1110-16 and EPM Dnr 2022-00012-02. For *LSR* the registration numbers are EPN Dnr 2016/179 and 2016/999.

Clinical variables and outcome
In Sweden, all hospitals treating patients with acute stroke report comprehensive data on for instance demographics, vascular risk factors, acute treatments and severity of the stroke, and outcomes to the national quality register for stroke, Riksstroke. Data are entered into this register by licensed healthcare personnel at each hospital, and the coverage rate is about 95% (www.riksstroke.org/general-information/). For both *SAHLSIS2* and *LSR* data on diabetes mellitus, hypertension, smoking at baseline and mortality within 3 months were obtained from Riksstroke. We also used data from Riksstroke on self-reported dependency in specified domains (mobility, dressing, and toileting), living conditions, and need of help or support from next of kin 3 months post-stroke to estimate the modified Rankin Scale (mRS) scores 0-2, 3, 4 or 5 using a validated translation algorithm. Good outcome was defined as independency (mRS 0-2) and poor outcome as dependency or death (mRS 3-6). For *SAHLSIS2* data on the admission stroke severity, i.e. National Institutes of Health Stroke Scale (NIHSS) score, was obtained from Riksstroke. For *LSR* the initial NIHSS score from admission, as close to day 1 as possible, was registered.

Single nucleotide polymorphism (SNP) intensity data

The Illumina Infinium Global Screening Array-24 v3.0 BeadChip was used for genotyping of both *SAHLSIS2* and *LSR*. The array contains 5,006 single nucleotide polymorphisms in the male specific part of the Y chromosome. The microarray intensity data of the Y chromosome were normalized against cluster files created from male samples only. The pre-processing of the data was accomplished using GenomeStudio, and clustering was performed based on the signal intensities of the male specific part of the Y chromosome. Samples with a call rate lower than 98% and SNPs with a gene call score < 0.15 were excluded. The normalization was performed at the SNP&SEQ Technology Platform in Uppsala, and a Nexus plugin was used to generate the copy number variation (CNV) calls.

Statistics

For the final multivariable regression model, we used the R-package missForest (v 1.5) to impute missing data from the clinical variables NIHSS scores and diabetes mellitus in order to retain power in the final model.

STROBE Statement—checklist of items that should be included in reports of cohort studies

	Item No	Recommendation	Reported on page
**Title and abstract**	1	(*a*) Indicate the study's design with a commonly used term in the title or the abstract	1
		(*b*) Provide in the abstract an informative and balanced summary of what was done and what was found	1
**Introduction**			
Background/rationale	2	Explain the scientific background and rationale for the investigation being reported	3
Objectives	3	State specific objectives, including any prespecified hypotheses	3
**Methods**			
Study design	4	Present key elements of study design early in the paper	3-4 and Supplement
Setting	5	Describe the setting, locations, and relevant dates, including periods of recruitment, exposure, follow-up, and data collection	3 and Supplement
Participants	6	(*a*) *Cohort study*—Give the eligibility criteria, and the sources and methods of selection of participants. Describe methods of follow-up<br>*Case-control study*—Give the eligibility criteria, and the sources and methods of case ascertainment and control selection. Give the rationale for the choice of cases and controls<br>*Cross-sectional study*—Give the eligibility criteria, and the sources and methods of selection of participants	3 and Supplement
		(*b*) *Cohort study*—For matched studies, give matching criteria and number of exposed and unexposed<br>*Case-control study*—For matched studies, give matching criteria and the number of controls per case	na
Variables	7	Clearly define all outcomes, exposures, predictors, potential confounders, and effect modifiers. Give diagnostic criteria, if applicable	3-4 and Supplement
Data sources/ measurement	8*	For each variable of interest, give sources of data and details of methods of assessment (measurement). Describe comparability of assessment methods if there is more than one group	3 and Supplement
Bias	9	Describe any efforts to address potential sources of bias	4
Study size	10	Explain how the study size was arrived at	na
Quantitative variables	11	Explain how quantitative variables were handled in the analyses. If applicable, describe which groupings were chosen and why	3-4
Statistical methods	12	(*a*) Describe all statistical methods, including those used to control for confounding	4
		(*b*) Describe any methods used to examine subgroups and interactions	4
		(*c*) Explain how missing data were addressed	4 and Supplement
		(*d*) *Cohort study*—If applicable, explain how loss to follow-up was addressed	na

		Case-control study—If applicable, explain how matching of cases and controls was addressed<br>*Cross-sectional study*—If applicable, describe analytical methods taking account of sampling strategy	
		(*e*) Describe any sensitivity analyses	4
**Results**			
Participants	13*	(a) Report numbers of individuals at each stage of study—eg numbers potentially eligible, examined for eligibility, confirmed eligible, included in the study, completing follow-up, and analysed	4 and Figure 1
		(b) Give reasons for non-participation at each stage	na
		(c) Consider use of a flow diagram	na
Descriptive data	14*	(a) Give characteristics of study participants (eg demographic, clinical, social) and information on exposures and potential confounders	Table 1
		(b) Indicate number of participants with missing data for each variable of interest	na
		(c) *Cohort study*—Summarise follow-up time (eg, average and total amount)	na
Outcome data	15*	*Cohort study*—Report numbers of outcome events or summary measures over time	Table 1
		Case-control study—Report numbers in each exposure category, or summary measures of exposure	na
		Cross-sectional study—Report numbers of outcome events or summary measures	na
Main results	16	(*a*) Give unadjusted estimates and, if applicable, confounder-adjusted estimates and their precision (eg, 95% confidence interval). Make clear which confounders were adjusted for and why they were included	Figure 1and Supplement Table S1
		(*b*) Report category boundaries when continuous variables were categorized	na
		(*c*) If relevant, consider translating estimates of relative risk into absolute risk for a meaningful time period	na
Other analyses	17	Report other analyses done—eg analyses of subgroups and interactions, and sensitivity analyses	4-5 and Figure 1
**Discussion**			
Key results	18	Summarise key results with reference to study objectives	5
Limitations	19	Discuss limitations of the study, taking into account sources of potential bias or imprecision. Discuss both direction and magnitude of any potential bias	6
Interpretation	20	Give a cautious overall interpretation of results considering objectives, limitations, multiplicity of analyses, results from similar studies, and other relevant evidence	5-6
Generalisability	21	Discuss the generalisability (external validity) of the study results	6
**Other information**			
Funding	22	Give the source of funding and the role of the funders for the present study and, if applicable, for the original study on which the present article is based	7

*Give information separately for cases and controls in case-control studies

Note: An Explanation and Elaboration article discusses each checklist item and gives methodological background and published examples of transparent reporting. The STROBE checklist is best used in conjunction with this article (freely available on the Web sites of PLoS Medicine at http://www.plosmedicine.org/, Annals of Internal Medicine at http://www.annals.org/, and Epidemiology at http://www.epidem.com/). Information on the STROBE Initiative is available at www.strobe-statement.org

Tables

Table S1. Baseline characteristics, LOY and functional outcome (mRS score) for male ischemic stroke patients in the non-recanalization therapy and the recanalization therapy groups in the exploratory (*SAHLSIS2*) and validation (*LSR*) cohorts.

	SAHLSIS2			LSR		
**Characteristics**	**All**	**Non-recanalization therapy group**	**Recanalization therapy group**	**All**	**Non-recanalization therapy group**	**Recanalization therapy group**
**n**	588	399	189	735*	588	142
**Age, years, median [IQR]**	71 [60-78]	70 [60-78]	71 [61-79]	74 [66-80]	73 [66-80]	75 [66-80]
**Initial NIHSS score, median [IQR]**	2 [0-6]	1 [0-3]	7.5 [3-15]	3 [2-7]	3 [1-5]	9 [5-16]
**Current smoking, n (%)**	62 (11)	48 (12)	14 (5)	83 (11)	71 (12)	12 (9)
**Diabetes mellitus, n (%)**	96 (16)	70 (18)	26 (14)	167 (22)	143 (24)	24 (17)
**Hypertension, n (%)**	307 (52)	211 (53)	96 (51)	426 (58)	341 (58)	84 (59)
**LOY, n (%)**	83 (14)	52 (13)	31 (17)	123 (17)	95 (16)	26 (18)
**mRS score >2, n (%)**	185 (32)	130 (33)	55 (29)	278 (38)	214 (36)	61 (43)

Data are shown as median and interquartile range (IQR) or number (n) and percentage.
NIHSS: National Institutes of Health Stroke Scale, LOY: loss of chromosome Y defined as mLRR-Y <-0.15, mRS: modified Rankin Scale.
*Data on recanalization therapy missing for n=5 in *LSR*
